# Comparison of cisplatin-induced anti-tumor response in CT26 syngeneic tumors of three BALB/c substrains

**DOI:** 10.1186/s42826-021-00110-3

**Published:** 2021-12-08

**Authors:** Jeong Eun Gong, You Jung Jin, Ji Eun Kim, Yun Ju Choi, Su Jin Lee, Kil Soo Kim, Young Suk Jung, Joon Yong Cho, Yong Lim, Hyun Gu Kang, Dae Youn Hwang

**Affiliations:** 1 Department of Biomaterials Science (BK21 FOUR Program), College of Natural Resources & Life Science/Life and Industry Convergence Research Institute/Laboratory Animals Resources Center, Pusan National University, Miryang, South Korea; 2 College of Veterinary Medicine, Kyungpook National University, Daegu, South Korea; 3 College of Pharmacy, Pusan National University, Busan, South Korea; 4 Exercise Biochemistry Laboratory, Korea National Sport University, Seoul, South Korea; 5 Department of Clinical Laboratory Science, College of Nursing and Healthcare Science, Dong-Eui University, Busan, South Korea; 6 Department of Veterinary Theriogenology, College of Veterinary Medicine, Chungbuk National University, Cheongju, South Korea

**Keywords:** BALB/c, BALB/cKorl, Substrains, Cisplatin, CT26 colon cancer cell

## Abstract

**Background:**

To determine whether the background of BALB/c substrains affects the response to anti-tumor drugs, we measured for alterations in tumor growth, histopathological structure of the tumor, and expressions of tumor-related proteins in three BALB/c substrains derived from different sources (BALB/cKorl, BALB/cA and BALB/cB), after exposure to varying concentrations of cisplatin (0.1, 1 and 5 mg/kg).

**Results:**

Cisplatin treatment induced similar responses for body and organ weights, serum analyzing factors, and blood analyzing factors in all BALB/c substrains with CT26 syngeneic tumor. Few differences were detected in the volume and histopathological structure of the CT26 tumor. Growth inhibition of CT26 tumors after exposure to cisplatin was greater in the BALB/cB substrain than BALB/cKorl and BALB/cA substrains, and a similar pattern was observed in the histopathological structure of tumors. However, the expression levels of other tumor-related factors, including Ki67, p27, p53, Bcl-2-associated X protein (Bax), B-cell lymphoma 2 (Bcl-2), caspase-3 (Cas-3), matrix metallopeptidase 2 (MMP2) and vascular endothelial growth factor (VEGF) proteins, were constantly maintained in the tumors of all three substrains after cisplatin treatment. A similar decrease pattern was observed for the expressions of inflammatory cytokines, including interleukin (IL)-1β, IL-6 and IL-10, in the CT26 tumors of the three BALB/c substrains.

**Conclusions:**

Taken together, results of the present study indicate that the genetic background of the three BALB/c substrains has no major effect on the therapeutic responsiveness of cisplatin, except growth and histopathology of the CT26 syngeneic tumor.

**Supplementary Information:**

The online version contains supplementary material available at 10.1186/s42826-021-00110-3.

## Background

BALB/c mice, derived from the Bagg albino strain provided by Halsey J. Bagg of the Memorial Hospital (NY, USA), are one of the well-known inbred strains widely used in immunology and cancer researches [1, 2]. These mice were first established as the stable BALB/cJ strain at The Jackson Laboratory in 1935 [2, 3]. Since then, the BALB/cByJ substrain was isolated from the BALB/cJ strain, based on the high reproductivity and low aggressiveness [4]. Furthermore, the BALB/cAnNCrl substrain was established from BALB/cJ and BALB/cByJ mice in the period 1950–1970 [4]. Recently, a new substrain (BALB/cKorl mice) was isolated from BALB/cJ, at the Department of Laboratory Animal Resources of the National Institute of Food and Drug Safety Evaluation (NIFDS, Chungju, Korea) [5].

Varied responses of some BALB/c substrains have been examined in several physiological fields. Different responses were detected in the genetic and environmental control of diabetes induced by multidose streptozotocines in BALB/cJ and BALB/cByJ substrains [6]. Furthermore, thirteen isolated lines established by full-sib inbreeding BALB/c mice showed differences in their reactions in the deficiency of the corpus callosum, while the BALB/cWah 1 line exhibited a spontaneous change after 7 generations of inbreeding [7]. The BALB/cJ and BALB/cAnNCr substrains were susceptible to Theiler’s murine encephalomyelitis virus (TMEV)-induced demyelinating disease, although the BALB/cByJ and BALB/cCum substrains showed resistance to this virus [8]. Furthermore, the BALB/cJ, BALB/cByJ and BALB/cAnNCr substrains showed varied aggressive behaviors [2, 4]. A significant difference was observed in the sensitivity for dexamethasone-induced osteonecrosis when comparing the BALB/cJ and BALB/cAnNHsd substrains [9]. The differences in skin tumor induction efficacy to DMBA and TPA cotreatment were also analyzed in three BALB/c substrains, including BALB/cKorl [5]. However, no study has examined the outcomes in the anti-tumor response to cisplatin in BALB/c substrains with syngeneic tumors.

The current study investigated the influence of genetic backgrounds on the anti-tumor response induced by cisplatin in CT26 syngeneic tumors of BALB/cKorl, BALB/cA and BALB/cB substrains.

## Results

### Response of BALB/c substrains on the growth and volume of cisplatin treated CT26 tumor

**Fig. 1:**
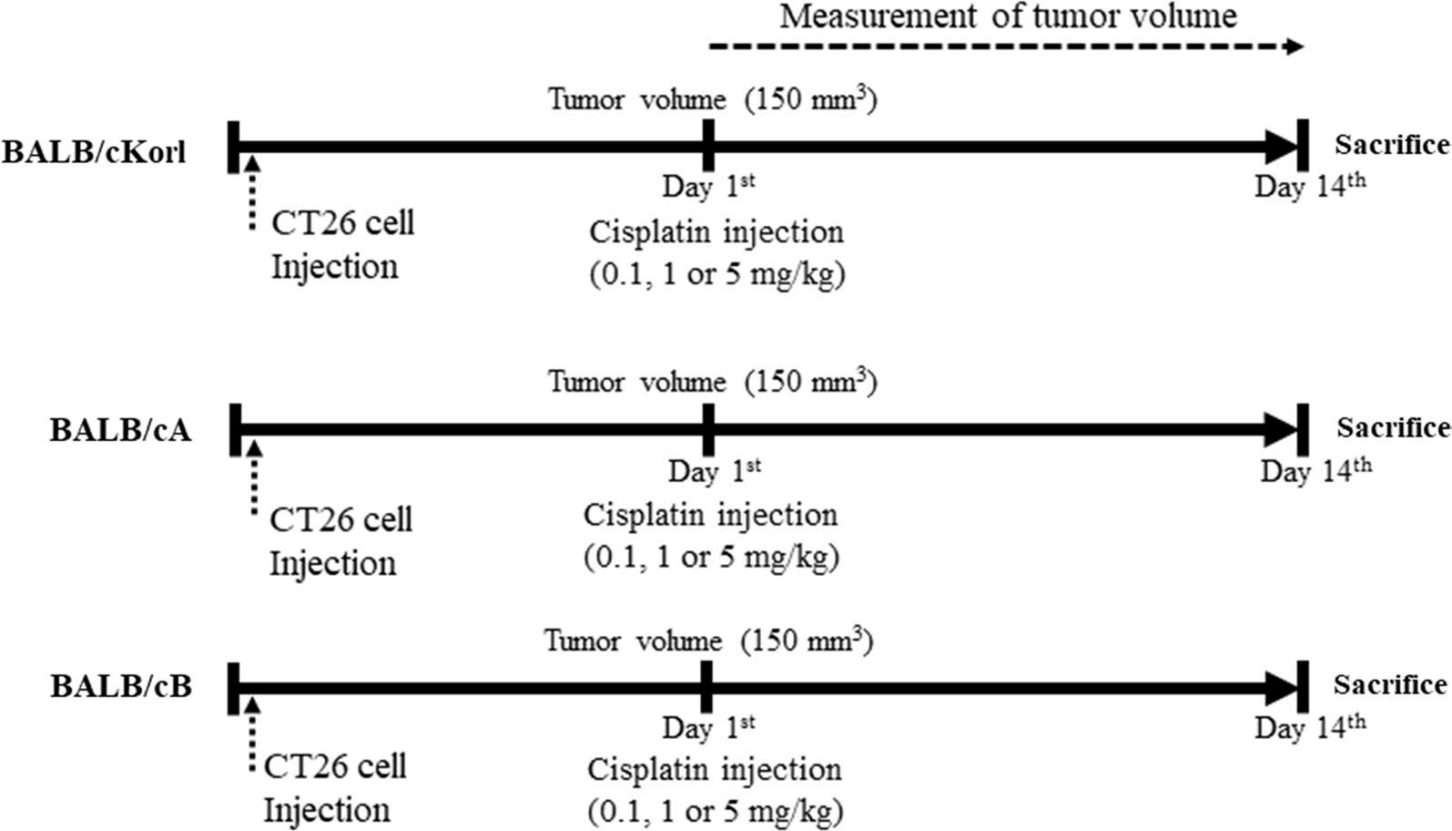
Experimental scheme for CT26 cell injection and cisplatin treatment in the three BALB/c substrains. After subcutaneous injection of CT26 cells, the syngeneic model was intraperitoneally administered varying doses of cisplatin (0.1, 1 and 5 mg/kg) once the tumor size reached 150 mm^2^

**Fig. 2:**
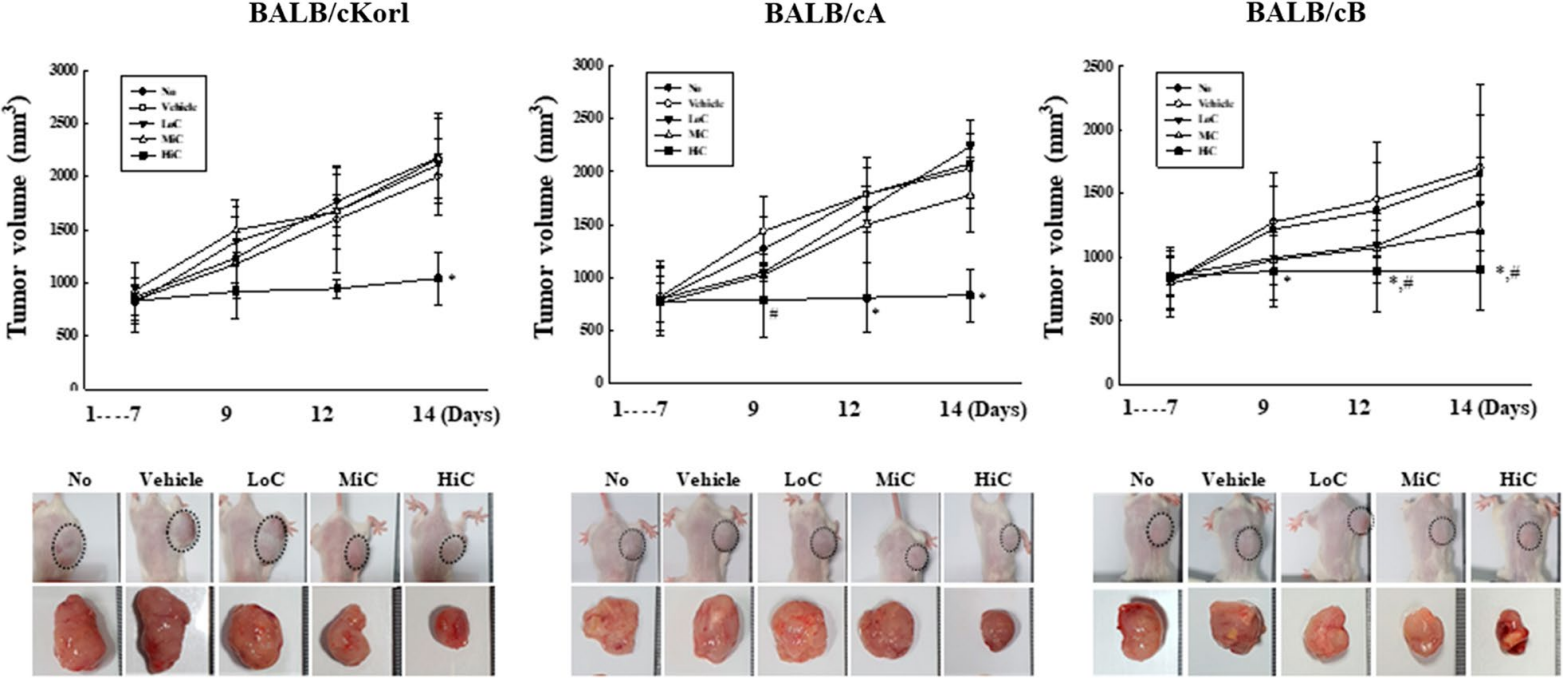
CT26 tumor volume in the three BALB/c substrains treated with cisplatin. The size of solid tumor formed in the syngeneic model was measured using a caliper from days 1 to 14, although significant changes were detected from day 7 to day 14. Five to six mice per group were used for measurement of tumor size; the volume of tumor was calculated in duplicate for each tumor. Data represent the mean ± SD. *, *p* < 0.05 compared to the No treated group. #, *p* < 0.05 compared to the Vehicle treated group

We first examined whether the background of BALB/c substrains affects the growth and volume of CT26 tumor after exposure to cisplatin. To achieve this, alterations in the growth and volume of CT26 syngeneic tumors were measured in three BALB/c substrains after cisplatin treatment for 14 days (Fig. 1). Of the three substrains examined, the BALB/cB response to cisplatin differed from the other two BALB/c substrains. Increase in rate of tumor volume was greater in the BALB/cKorl and BALB/cA substrains than BALB/cB substrain, although the increase over time was gradual in all three. However, the volumes were significantly decreased in all HiC treated groups of the three BALB/c substrains. Especially, tumor volume in LoC and MiC treated groups were significantly decreased only in BALB/cB, but not in the BALB/cKorl and BALB/cA substrains (Fig. 2). However, the body and organ weight, and the levels of serum and blood analyzing factors were consistently maintained in three BALB/c substrains (Additional file 1: Tables S1, S2 and S3). These results indicate that anti-tumor effects of cisplatin on the growth and volume of CT26 syngeneic tumors are affected by the background of BALB/c substrains. In addition, this study further showed that sensitivity to the anti-tumor effect of cisplatin is associated with the magnitude of CT26 syngeneic tumor volume in three BALB/c substrains.

### Effects of BALB/c substrains on the histopathological structure of cisplatin treated CT26 tumor

**Fig. 3:**
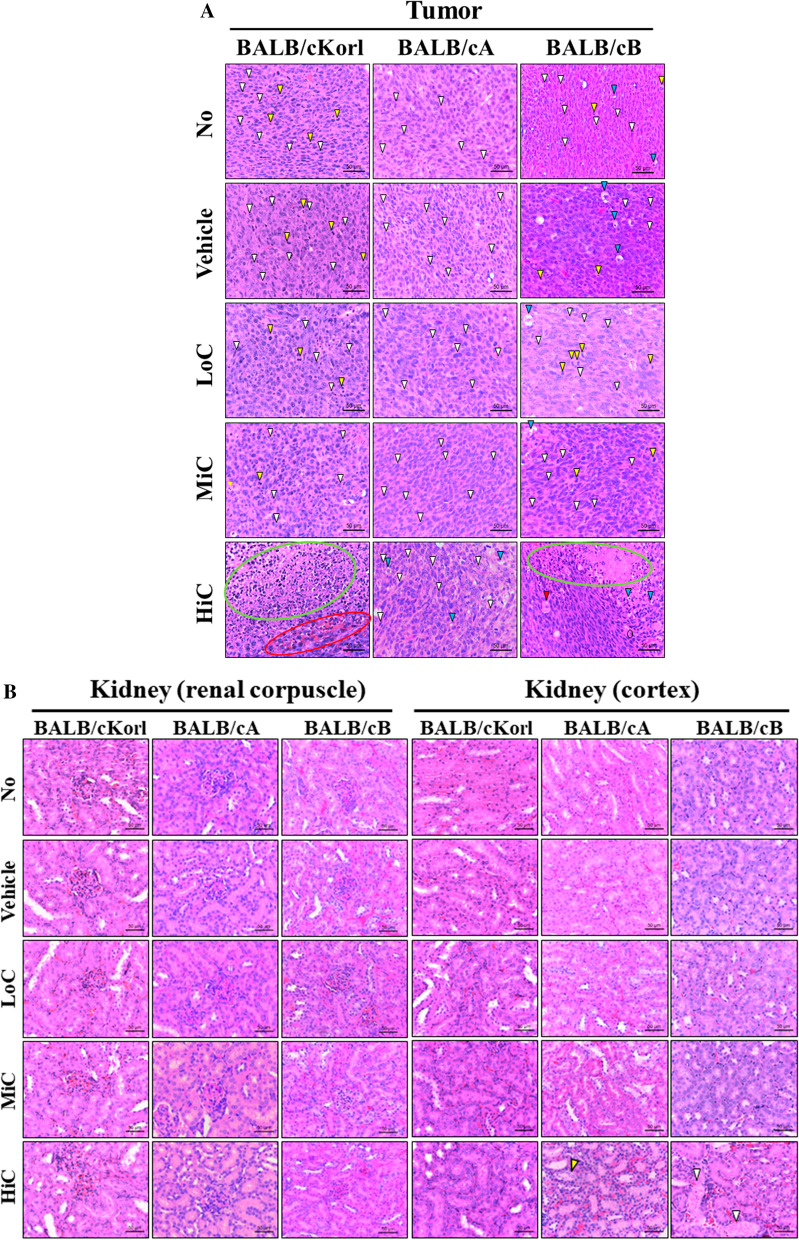

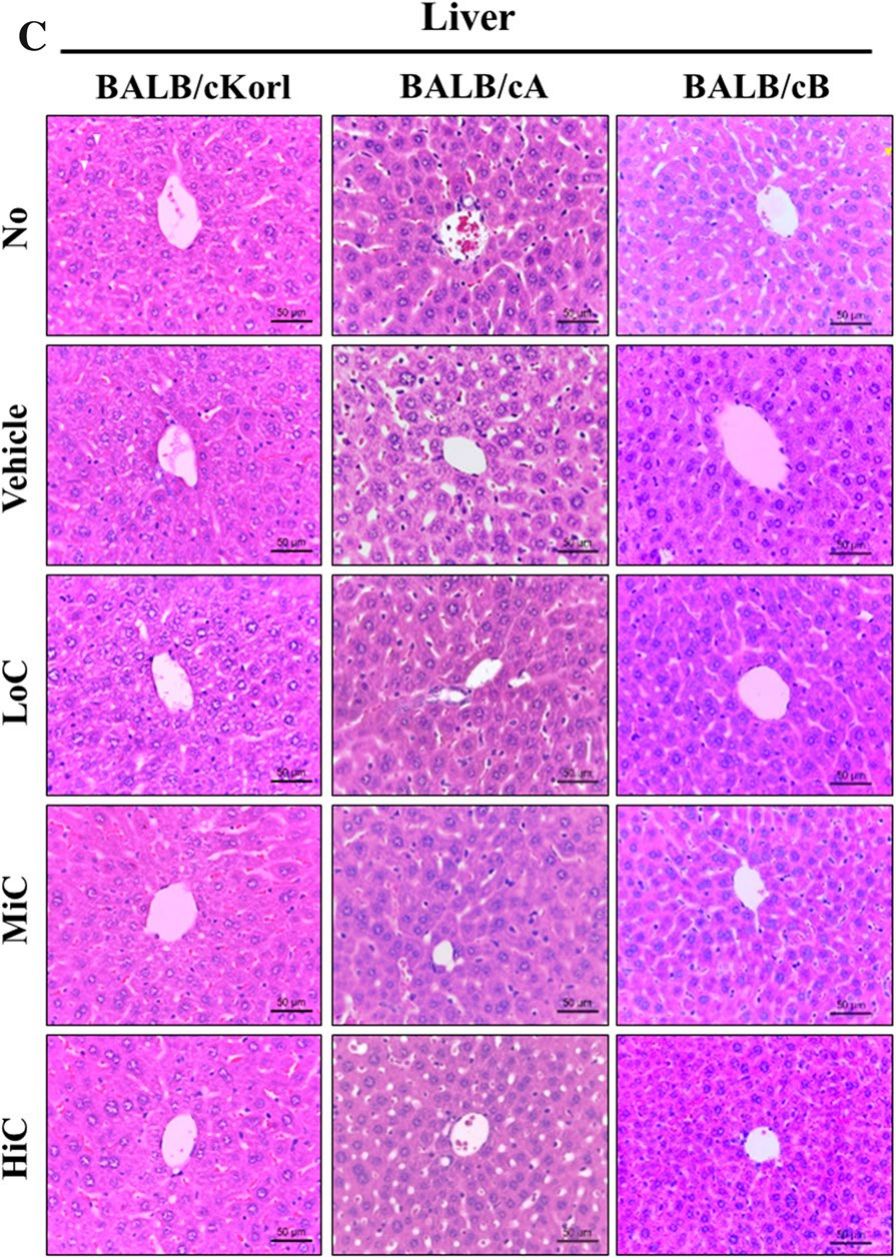
The histopathological structure of CT26 tumor, kidney and liver. **a** Tumorigenic changes. After harvesting the CT26 tumors from the three BALB/c substrains, the histopathological images were obtained at 400 × magnification, from slide sections of tumor tissue stained with H&E solution. Various tumorigenic changes, such as spindle cells (white arrow), mitotic figures (yellow arrow), vacuolated tumor cells (blue arrow), hemorrhage (red circle), necrosis (green circle) and plumped cell (red arrow), were characterized by a pathologist, Dr. Sang Gu Lee. **b** Histopathological structure of kidney. Various tumorigenic changes, such as necrosis (white arrow) and tubular bashophilia (yellow arrow), were characterized by a pathologist, Dr. Sang Gu Lee. **c** Histopathological structure of liver. Five to six mice per group were used for preparation of tissue sections and H&E staining, and histopathological structure was analyzed in duplicate for each tumor

To investigate the background effect of different BALB/c substrains on the histopathology of CT26 tumor after cisplatin treatment, we examined for alterations in the histopathological structure of CT26 tumors in three syngeneic BALB/c substrains after 14 days exposure to cisplatin. Changes in the histopathological structure of CT26 tumor were more severe in the BALB/cB substrain than the BALB/cKorl and BALB/cA substrains. Vehicle, LoC and MiC treated groups of the BALB/cKorl and BALB/cA substrains exhibited a solid pattern containing spindle cells, whereas the same groups in the BALB/cB substrain showed a solid pattern containing vacuolated tumor cells, mitotic pattern and spindle cells (Fig. 3a and Table 1). Similar differences were observed in the HiC treated group. A solid pattern involving hemorrhage and severe necrosis was detected in the CT26 tumor of the BALB/cKorl substrain, whereas mixed type tumors, including vacuolated tumor cells and spindle cells, were increased in the BALB/cA substrains. However, the BALB/cB substrain included a large number of plumped cells, severe necrosis, hemorrhage and vacuolated tumor cells in the histopathological structure of the CT26 tumor (Fig. 3a). Also, any significant pathological alterations were not observed in the kidney and liver section (Fig. 3b, c). Taken together, these results indicate that anti-tumor effects of cisplatin on the histopathological structure of CT26 tumors are affected by the type of BALB/c substrain. Our results further suggest that the histopathological structure of CT26 tumors in the BALB/cB substrain is more sensitive to cisplatin exposure, as compared to the BALB/cKorl and BALB/cA substrains.

**Table 1:** Histopathological structure of CT26 tumor and kidney

Mice	BALB/cKorl	BALB/cA	BALB/cB
Tumor			
Vehicle, LoC and MiC treated group	Solid pattern including spindle cells and mitotic pattern	Solid pattern including spindle cells	Solid pattern including vacuolated tumor cells, mitotic pattern and spindle cells
HiC treated group	Solid pattern including hemorrhage and severe necrosis	Mixed type including vacuolated tumor cells and spindle cells	Mixed type including plumped cells, severe necrosis, hemorrhage and vacuolated tumor cells
Kidney			
Vehicle, LoC and MiC treated group	Normal	Normal	Normal
HiC treated group	Normal	Focal, minimal necrosis	Focal, minimal, tubular bashophilia

### Effects of BALB/c substrains on the cell proliferation ability of cisplatin treated CT26 tumor

**Fig. 4:**
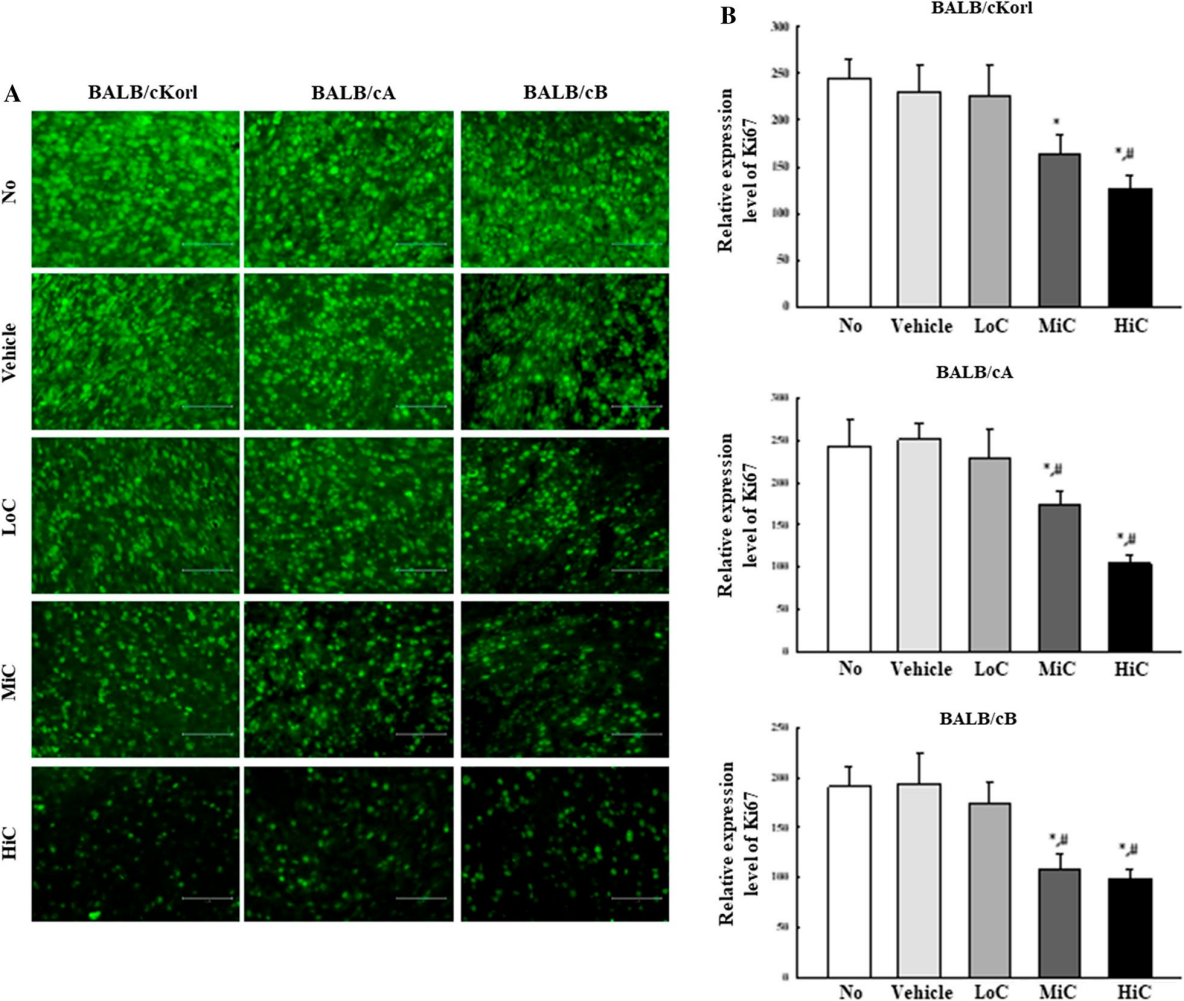
IF assays for Ki67 protein. **a** After IF staining, the green fluorescence was observed at a fluorescent microscope. **b** The number of Ki67 positive cells were measured within 67,500 μm^2^ of the CT26 tumor section, and results are shown as a percentage of control. Two to three tumors per group were used in the preparation of the Ki67 stained sample, and the number of Ki67 positive cells was counted in duplicate for each sample. The data are reported as the means ± SD. *, *p* < 0.05 relative to the No treated group. #, *p* < 0.05 compared to the Vehicle treated group

To investigate whether background of the BALB/c substrain affects the cell proliferation ability of CT26 tumor after cisplatin treatment, immunofluorescence (IF) intensity for the Ki67 protein was measured on CT26 tumor sections obtained from the three syngeneic BALB/c substrains after exposure to cisplatin for 14 days. A slight dose-dependent decrease was observed in the LoC, MiC and HiC treated groups (Fig. 4a, b). These patterns were maintained in all three BALB/c substrains, indicating that background of the BALB/c substrain does not majorly affect alterations on the expressions of Ki67 proteins for cell proliferation in the cisplatin treated CT26 tumors.

### Effects of BALB/c substrain on the tumor suppressing ability of cisplatin treated CT26 tumor

**Fig. 5:**
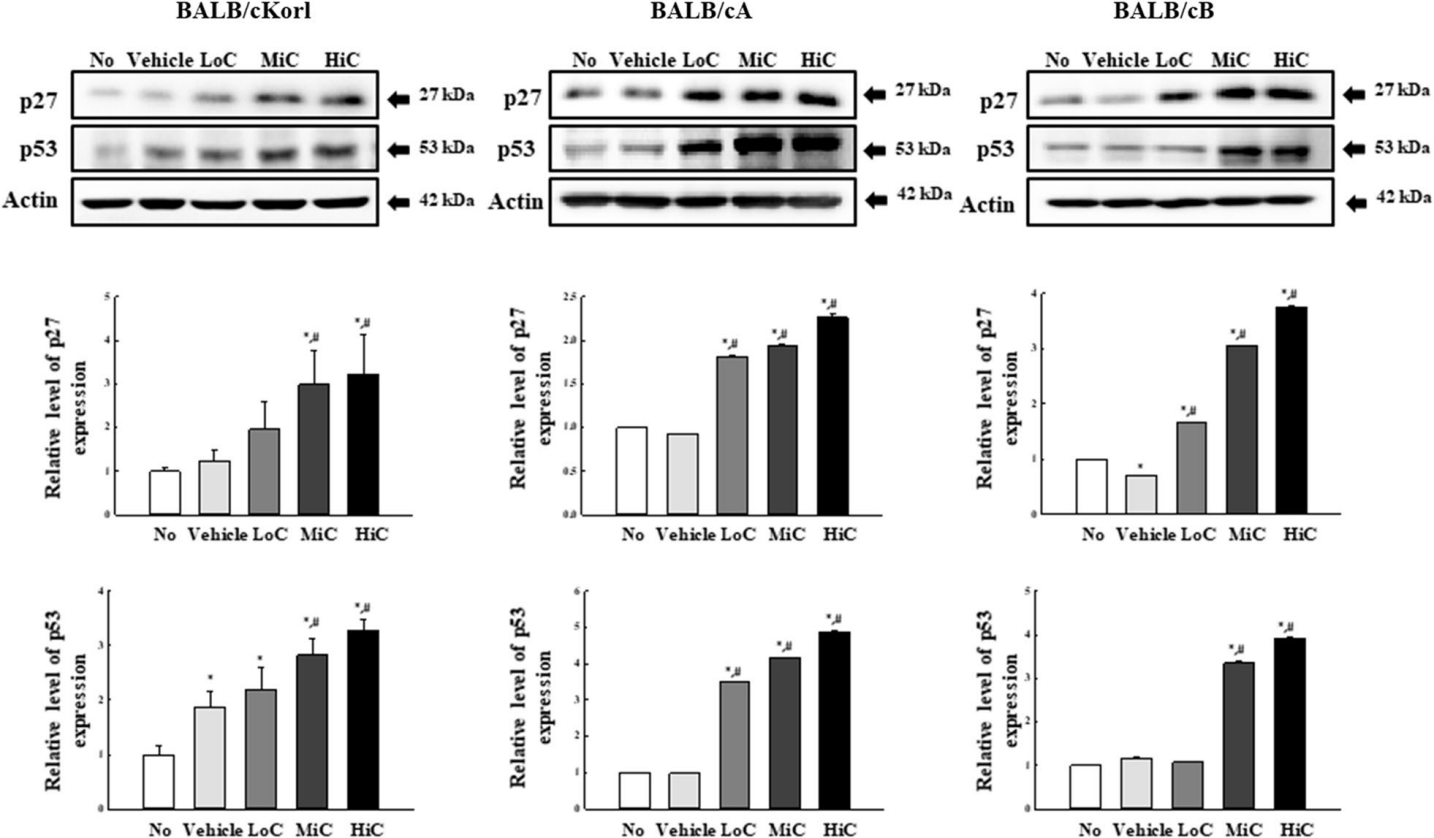
Expression analysis of p53 and p27 proteins. Expression levels of p53 and p27 proteins were determined in CT26 tumors of the three cisplatin-treated BALB/c substrains, using specific antibodies and an imaging densitometer. The levels of each protein are presented relative to the intensity of actin. Two to three tumors per group were used to prepare the total tumor homogenate, and Western blot analysis was assayed in duplicate for each sample. The data are reported as the means ± SD. *, *p* < 0.05 relative to the No treated group. #, *p* < 0.05 compared to the Vehicle treated group

To investigate whether background of the BALB/c substrain affects the tumor suppressing ability of CT26 tumor after cisplatin treatment, we measured the expression levels of p27 and p53 proteins in CT26 syngeneic tumors obtained from the three BALB/c substrains after cisplatin treatment for 14 days. Protein expression levels were remarkably and dose-dependently increased after cisplatin exposure. The increase patterns were similar for all three BALB/c substrains, although few variations were observed for the total amount of proteins (Fig. 5). These results indicate that background of the three BALB/c substrains does not majorly affect alterations in the expressions of p27 and p53 proteins in the cisplatin treated CT26 syngeneic tumors.

### Effects of BALB/c substrains on the apoptotic process in cisplatin treated CT26 tumor

**Fig. 6:**
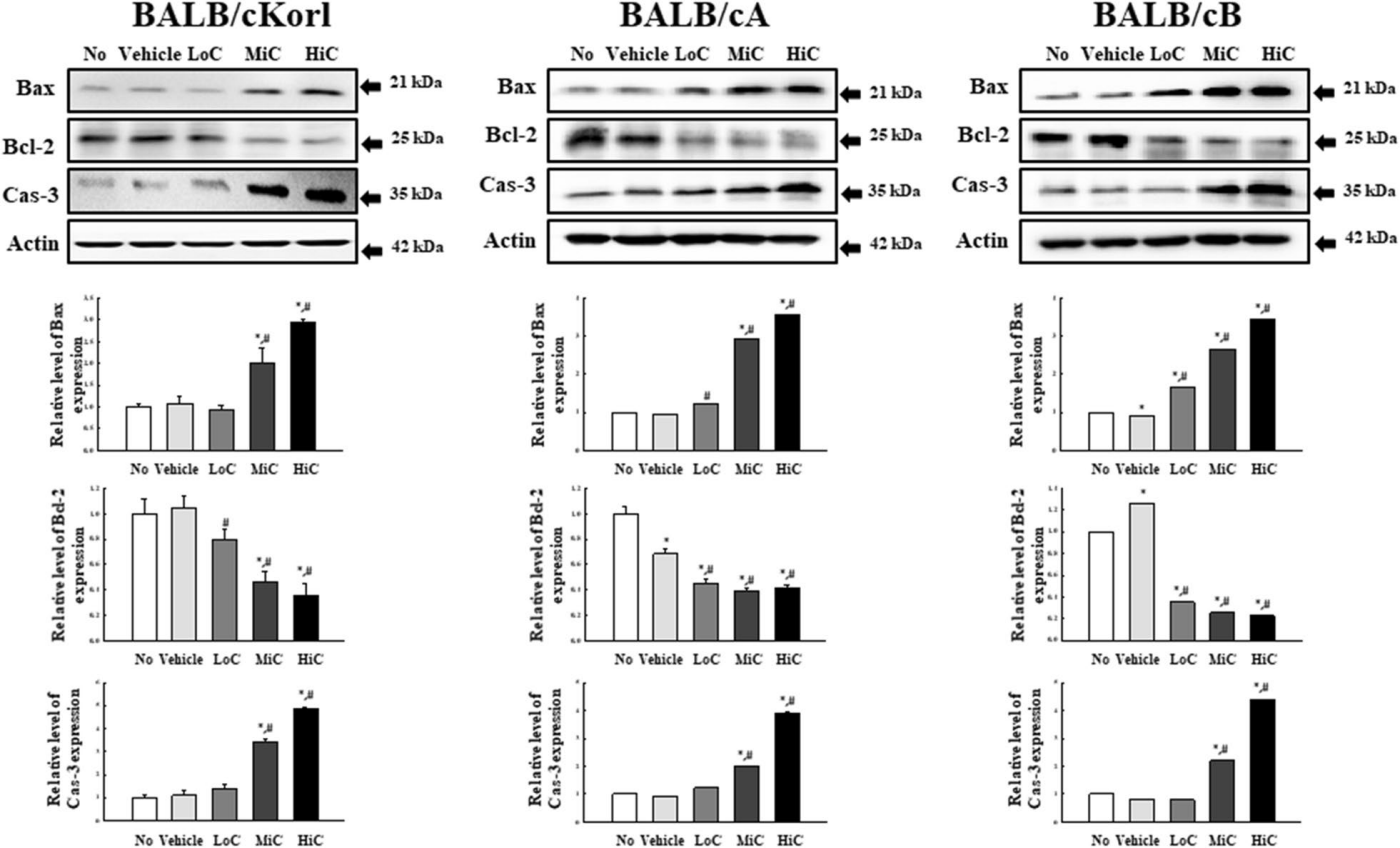
Expression analysis of Bax, Bcl-2 and Cas-3 proteins. Expression levels of Bax, Bcl-2 and Cas-3 proteins were determined in CT26 tumors of the three cisplatin-treated BALB/c substrains, using the specific antibodies and an imaging densitometer. Levels of each protein are presented relative to the intensity of actin. Two to three tumors per group were used to prepare the total tumor homogenate, and Western blot analysis was assayed in duplicate for each sample. The data are reported as the means ± SD. *, *p* < 0.05 relative to the No treated group. #, *p* < 0.05 compared to the Vehicle treated group

To investigate whether background of the BALB/c substrain affects the apoptosis of CT26 tumor after cisplatin treatment, the expression levels of Bax, Bcl-2 and Cas-3 proteins were measured in CT26 syngeneic tumors obtained from the three BALB/c substrains after 14 days exposure to cisplatin. A dose-dependent increase was observed in the expression levels of Bax and Cas-3 proteins, whereas a reverse pattern was observed for Bcl-2 protein expression. However, the expression patterns were similar in all three BALB/c substrains, although there were a few variations for the total amount of each protein (Fig. 6). The above results indicate that the apoptotic process is not majorly affected by the background BALB/c substrains in cisplatin treated CT26 syngeneic tumors of BALB/cKorl, BALB/cA and BALB/cB substrains.

### Effects of BALB/c substrains on the metastatic ability of cisplatin treated CT26 tumor

**Fig. 7:**
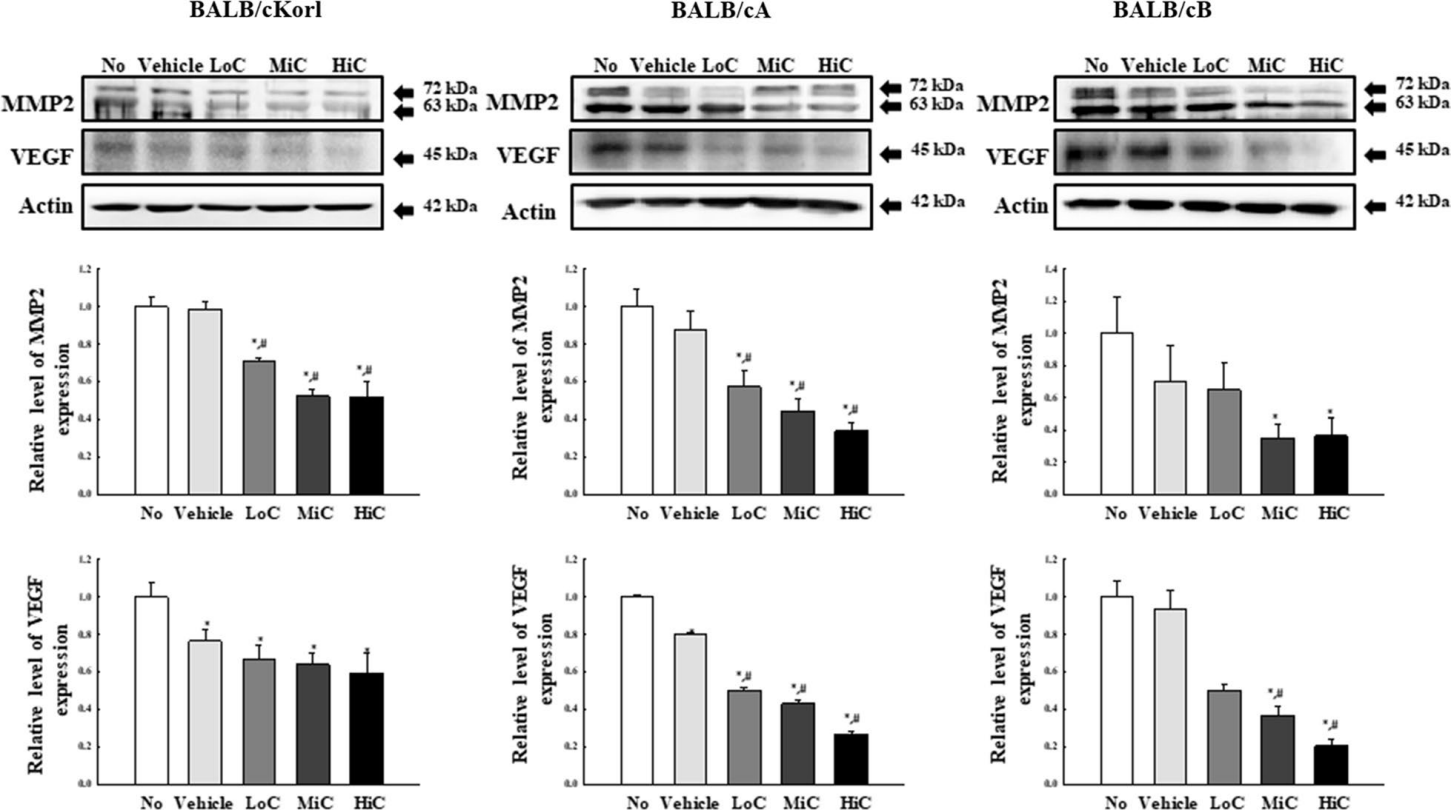
Expression analysis of MMP2 and VEGF protein. Expression levels of MMP2 and VEGF protein were determined in CT26 tumors of the three cisplatin-treated BALB/c substrains, using the specific antibodies and an imaging densitometer. Levels of each protein are presented relative to the intensity of actin. Two to three tumors per group were used to prepare the total tumor homogenate, and Western blot analysis was assayed in duplicate for each sample. The data are reported as the means ± SD. *, *p* < 0.05 relative to the No treated group. #, *p* < 0.05 compared to the Vehicle treated group

To investigate whether background of the BALB/c substrain affects the metastatic ability of the CT26 tumor after cisplatin treatment, the expression levels of MMP2 and VEGF proteins were examined in CT26 syngeneic tumors obtained from the three BALB/c substrains after cisplatin treatment for 14 days. Expression levels of both proteins were dose-dependently inhibited after cisplatin treatment, and were similar in all three BALB/c substrains, with some variation in total amount of each protein (Fig. 7). These results indicate that background of the three BALB/c substrains has no major effect on the metastatic ability of the CT26 syngeneic tumors obtained from BALB/cKorl, BALB/cA and BALB/cB substrains after cisplatin treatment.

### Effects of BALB/c substrains on the inflammatory response of cisplatin treated CT26 tumors

**Fig. 8:**
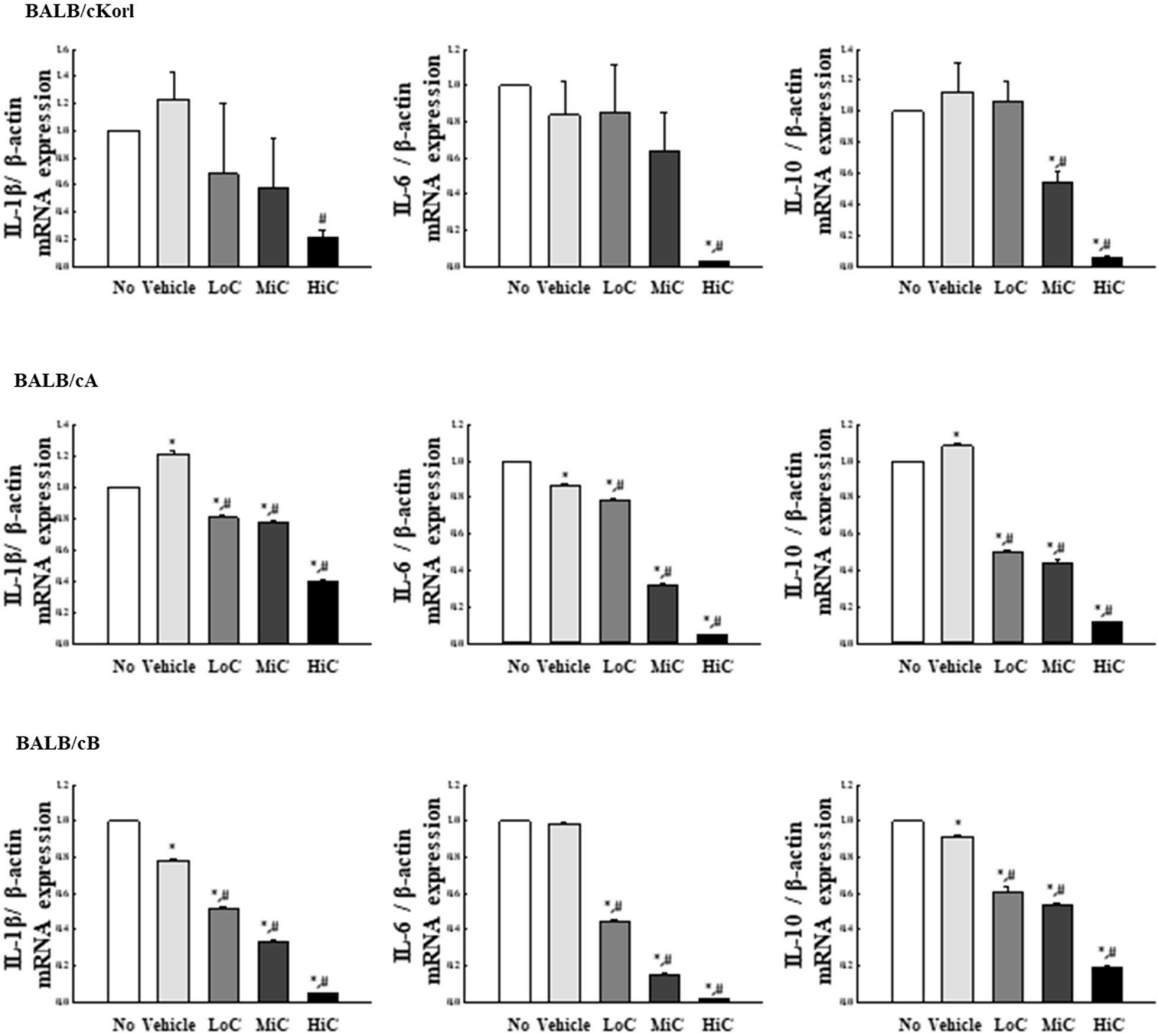
Transcription level of inflammatory cytokines. After collection of total RNA from CT26 tumor of the three cisplatin-treated BALB/c substrains, the mRNA levels of IL-1β, IL-6 and IL-10 genes were measured by quantitative realtime – polymerase chain reaction (RT-qPCR), as described in materials and methods. Two to three tumors per group were used to prepare the total RNA, and RT-qPCR analysis was assayed in duplicate for each sample. The data are reported as the means ± SD. *, *p* < 0.05 relative to the No treated group. #, *p* < 0.05 compared to the Vehicle treated group

Finally, we investigated whether background of the BALB/c substrains affects the inflammatory response of CT26 syngeneic tumor after cisplatin treatment. The mRNA levels of three cytokines, viz., IL-1β, IL-6 and IL-10, were measured in CT26 tumors obtained from the three BALB/c substrains after cisplatin treatment for 14 days. A similar regulation pattern was observed for all three substrains. The mRNA levels of IL-1β, IL-6 and IL-10 were dose-dependently inhibited in the CT26 tumors (Fig. 8). These results indicate that alterations in the inflammatory response in the cisplatin treated CT26 syngeneic tumors of BALB/cKorl, BALB/cA and BALB/cB substrains are not affected by the BALB/c substrain background.

## Discussion

The different cell line-derived syngeneic tumor models (CDX) are produced by transplanting immortal cells isolated from tumors of the same species [10]. These mice are widely used to assess the efficacy of immuno-oncologic drugs, as well as therapeutic effects of anti-tumor drugs on the immune system [11]. During generation of these models, a specific genetic background of the recipient animal is considered an important factor that affects tumor development [11]. This study therefore investigated whether genetic background of BALB/c substrains can affect the anti-tumor response to cisplatin treatment in the syngenic model with CT26 tumor. To achieve this, the anti-tumor response of cisplatin was compared in three BALB/c substrains with CT26 syngeneic tumor. Our results indicate that most anti-tumor responses to cisplatin were very similar across the CT26 tumors of all three BALB/c substrains, although variations were detected in the growth and histopathological structure of these tumors.

Variations in responses of the BALB/c substrains in tumor studies have rarely been examined. One study compared the tumor incidence in two substrains (916 and 917) of Claude BALB/c (BALB/cfCd) mice. They reported that several tumors, including mammary tumor, neoplasms of the reticulo-endothelial system, and tumors of the respiratory system, had a higher incidence in the 917 family than 916 family, whereas renal tumors and synovial tumors showed the opposite tendency [12]. Similar differences in the BALB/c substrain were observed in the sensitivity to DMBA and TPA. The BALB/cJ substrain showed high level (64–100%) of tumor induction efficacy, whereas the BALB/cByJ substrain exhibited intermediate induction (42–58%) [13]. Differences in the tumor induction efficacy response to DMBA and TPA were examined in BALB/cKorl, BALB/cA and BALB/cB substrains, and were determined to be higher in the BALB/cKorl substrain than the other two substrains. However, other tumor phenotypes, including histopathology, apoptotic protein levels and tumor-related protein levels, were similarly induced. These patterns were likewise observed in the anti-tumor response to cisplatin treatment [5]. In the current study, we examined for variations in the anti-tumor response of cisplatin in three BALB/c substrains bearing CT26 syngeneic tumors. Our findings for CT26 tumor growth in response to cisplatin were similar to previous studies, which reported a higher level of tumor induction efficacy in the BALB/cKorl substrain after DMBA and TPA treatment. Especially, the dose-dependent response of cisplatin on tumor growth was clearly observed in the BALB/cB substrain, as compared to BALB/cKorl and BALB/cA substrains. Hence, results of the present study provide additional evidence for different responses between BALB/c substrains after exposure to an anti-tumor drug.

Cisplatin is a well-known chemotherapeutic agent used in the treatment of numerous tumors including brain, lung, esophageal, ovarian, breast, and bladder carcinomas, although its adverse effects induce the inhibition of bone marrow, kidney damage, vomiting, and hearing defects [14, 15]. During exposure to cisplatin, one of the most important mechanisms is induction of severe oxidative stress in the mitochondria, leading to apoptosis through the regulation of intrinsic and extrinsic pathways [16, 17]. Also, cisplatin-induced apoptosis is involved in the regulation of protein kinase c (PKC), MAPK signaling pathway, and PI3K/Akt signaling pathway [18–20]. Furthermore, cisplatin treatment induces an increase in the expression levels of p53 protein and some p53 transactivated proteins, including  Mouse double minute 2 (MDM2) and p21, in various tumor cells [21]. Cisplatin was the anti-tumor drug of choice in the current study, because the anti-tumor properties and mechanisms are well known in various cell lines and animal models. A significant increase in the p53 expression level was detected in cisplatin treated groups of all three BALB/c substrains. These results are very similar to previous studies, although the concentrations of treated cisplatin used were varied.

## Conclusions

The current study investigated whether the genetic background of BALB/c substrain affects the anti-tumor response of cisplatin in the CT26 syngeneic tumor model. Results of the present study indicate that all three BALB/c substrain CT26 syngenic models have an overall similar functionality and reactivity to cisplatin and anti-tumor drugs, barring few differences observed in the growth and histopathological structure of the tumor. Our results further validate that to evaluate the therapeutic effects of anti-tumor drugs, the BALB/cKorl substrain established in NIFDS can replace BALB/c substrains from other commercial suppliers. However, additional studies are required to expand our understanding of the therapeutic effects and molecular mechanism of various anti-tumor drugs with different efficacies.

## Methods

### Cell culture

CT26 is a well-known cell line derived from the colon tissue of tumor-bearing BALB/c mice, resulting from the implantation of primary CT26 cells. The cell line was procured from the ATCC (Cat. No. CRL-2638, Manassas, VA, USA). Cells were cultured in a humidified incubator at 37°C under 5% CO_2_ and 95% air, in Roswell Park Memorial Institute 1640 Medium (RPMI 1640 Medium, Cat. No. LM011-01, Welgene, Gyeongsan-si, Korea) supplemented with 10% fetal bovine serum (FBS), 2 mM glutamine, 100 U/mL penicillin, and 100 μg/mL streptomycin.

### Care and management of BALB/c mice

Experimental protocol for the syngeneic tumor model was carefully reviewed based on the ethical and scientific care guidelines, and approved by the Pusan National University-Institutional Animal Care and Use Committee (Approval No. PNU-2020-2656). A statistically significant number of mice were used to ensure reliability of the results in our experiments. Three male BALB/c substrains (7-weeks-old) were obtained from three different sources. The BALB/cKorl mice were kindly provided by the Department of Laboratory Animal Resources of the NIFDS (Chungju, Korea). The other two strains (BALB/cA and BALB/cB) were purchased from vendors located in the United States (Vendor A) and Japan (Vendor B), respectively. All mice were kept in solid-bottom cages with wood shavings, and provided ad libitum access to water and an irradiated standard chow diet (Samtako BioKorea Co., Osan, Korea). During the experimental period, all animals were maintained in a specific pathogen-free (SPF) environment under a strict light cycle (lights on at 08:00 A.M. and off at 08:00 P.M.), at 23 ± 2°C temperature and 50 ± 10% relative humidity. All mice were housed at the Pusan National University-Laboratory Animal Resources Center (PNU-LARC), which is accredited by the Korea Ministry of Food and Drug Safety (MFDS; Accredited Unit 000231) and Association for Assessment and Accreditation of Laboratory Animal Care (AAALAC) International (Accredited Unit 001525).

### Animal experiment for syngeneic tumor model

Briefly, CT26 cells (5 × 10^5^ cells) were subcutaneously injected into the dorsal region of BALB/cKorl (*n* = 40), BALB/cA (*n* = 40) or BALB/cB (*n* = 40) substrains at day 1. After the tumor attained a size of about 150 mm^3^ (day 1), the three CT26 tumor-bearing substrains were randomly divided into one of five groups (*n* = 8/group): (1) No treated group (*n* = 8), (2) Vehicle treated group (Vehicle, *n* = 8), constant volume of 1 × PBS every 3.5 days from day 1 to day 14; (3) low dose cisplatin treated group (LoC, *n* = 8), intraperitoneal injection of cisplatin (0.1 mg/kg), every 3.5 days from day 1 to day 14; (4) medium dose cisplatin treated group (MiC, *n* = 8), intraperitoneal injection of cisplatin (1 mg/kg), every 3.5 days from day 1 to day 14; (5) high dose cisplatin treated groups (HiC, *n* = 8), intraperitoneal injection of cisplatin (5 mg/kg), every 3.5 days from day 1 to day 14 (Fig. 1). At 24 h after the final treatment, all mice of subset groups were euthanized using a euthanasia chamber filled with CO_2_ gas, after which the solid tumors were collected from the dorsal region of mice. To prevent pain or distress of mice, the humane endpoint was set when the tumor exceeded 3,000 mm^3^ in volume, or when sudden decrease in body weight of mice was more than 10% within 1–2 weeks.

### Measurement of tumor volume and weight

Alteration in the volume of CT26 tumors in the BALB/c substrains was observed from days 1 to 14, including the period of cisplatin injection. Briefly, the length and width of tumors were measured by external calipers (Matusutoyo, Tokyo, Japan), and volume of each tumor was calculated using the following formula:

$${\text{Tumor ~volume~ (mm}}^3) = (A)*(B^2)/2$$

where A is the length of tumor (mm), and B is the width of tumor (mm).

The weight of each tumor harvested from syngeneic mice was measured using an electrical balance in duplicate (Mettler Toledo, Greifensee, Switzerland).

### Whole blood and serum analysis

After the experimental process, all mice were fasted for 8 h, following which anesthesia was induced by intravenous injection of Alfaxan (JUROX Pty Limited, Rutherford, Australia, 13 mg/kg body weight i.v.), and blood was subsequently collected from the abdominal veins using a 1 mL syringe attached to a needle (26 SWG). Blood analysis and serum biochemistry were performed for all collected samples. Whole blood was placed in plain capped bottles containing ethylenediaminetetraacetate (EDTA), and the components were analyzed using an automated cell counter (Beckman-Coulter Inc., Miami, FL, USA) with standard calibration, according to the manufacturer’s instructions. The levels of white blood cells (WBC), red blood cells (RBC), hemoglobin (HGB), hematocrit (HCT), mean corpuscular volume (MCV), mean corpuscular hemoglobin (MCH), mean corpuscular hemoglobin concentration (MCHC), corpuscular hemoglobin concentration mean (CHCM), corpuscular hemoglobin content (CH), hemoglobin concentration distribution width (HDW), platelets (PLT), and mean platelet volume (MPV) were measured in duplicate for each sample. Serum was obtained for biochemical analysis by centrifuging the whole blood at 1500 × g for 15 min. Serum biochemical components, including alkaline phosphatase (ALP), alanine aminotransferase (ALT), aspartate aminotransferase (AST), calcium (Ca) and low density lipoprotein (LDH), were assayed using an automatic serum analyzer (Hitachi 747; Hitachi, Tokyo, Japan). All assays were measured in duplicate using fresh serum.

### Histopathological analysis

Tumor, liver and kidney were harvested from CT26 tumor bearing BALB/c substrains of each subset group, and fixed in 10% formalin solution for 48 h. Fixed tissues were embedded into paraffin blocks after trimming and sectioned into 4 µm thick slices. The tumor, kidney and liver sections were then stained with hematoxylin and eosin (H&E) solution (Sigma-Aldrich, Merck KGaA, Darmstadt, Germany), and microscopically examined at 400 × magnification for histopathological features. The tumor type and pathological features of all tissues were characterized by a pathologist, Dr. Sang Gu Lee at DD Partner Co. (Seoul, Korea). Moreover, the necrotic area was measured and quantitated on H&E stained tumor sections, as described previously [22].

### IF staining analysis

CT26 tumor tissues collected from the syngeneic mice model were fixed in 10% formalin solution for 48 h. The central region of the solid tumor was embedded into paraffin blocks after trimming, and tissue blocks were then sectioned into 2 µm thick slices. The sections were deparaffinized, and subsequently hydrated in a graded series of ethanol solutions with a decreasing concentration. For antigen retrieval, the sections were heated for 30 min in sodium citrate buffer (10 mM, 0.05% tween 20, pH 6.0) at 95°C, followed by cooling for 20 min at room temperature. After washing three times with dH_2_O for 5 min, the slides were incubated with 5% bovine serum albumin (BSA) and 0.3% Triton in 1 × PBS for 1 h at room temperature, after which the slides were incubated overnight with the primary polyclonal antibody against rabbit Ki67/MKI67 (NB500-170 s, Novus Biologicals, Centennial, CO, USA) (1:100) in 1% BSA and 0.3% Triton in 1 × PBS at 4°C. Probed sections were washed three times with 0.5% Triton in 1 × PBS for 5 min, followed by incubation with secondary antibodies (1:100) in 1% BSA and 0.3% Triton in 1 × PBS for 1 h at room temperature. Sections were viewed and digitally photographed using an EVOS™ M5000 Imaging System (AMF5000, Thermo Fisher Scientific Inc., Bothell, WA, USA).

### Western blot analyses

Total proteins from CT26 tumors were prepared using the Pro-Prep Protein Extraction Solution (Cat. No. 17081, Intron Biotechnology Inc., Seongnam, Korea). Protein homogenates were collected after centrifugation at 13,000 rpm for 5 min, and the total protein concentration of each sample was determined using the SMART™ Bicinchoninic Acid Protein Assay Kit (Cat. No. 23225, Thermo Fisher Scientific Inc.). Total proteins (30 μg) were electrophoresed on 4–20% SDS-PAGE for 2 h, and subsequently transferred to 0.45 μm pore size nitrocellulose blotting membranes (Cat. No. 10600003, GE Healthcare, Little Chalfont, UK) for 2 h at 40 V. The membranes were subsequently incubated separately with the specific primary antibodies (Additional file 1: Table S4). Probed membranes were then washed with standard washing buffer, followed by incubation with 1:1,000 diluted horseradish peroxidase (HRP)-conjugated goat anti-rabbit IgG (Cat. No. G21234, Invitrogen, california, USA) for 1 h. Each protein blotted membrane was developed using the Amersham™ ECL Select™ Western Blotting detection reagent (Cat. No. RPN2235, GE Healthcare). Finally, the chemiluminescence signals derived from specific protein bands were measured using FluorChemi^®^FC2 (Alpha Innotech Co., San Leandro, CA, USA).

### RT-qPCR analysis

Frozen CT26 tumor tissues were homogenized using a Polytron PT-MR 3100 D Homogenizer (Kinematica AG, Lusern, Switzerland) in RNA Bee solution (Tet-Test Inc., Friendswood, TX, USA), based on the manufacturer’s instructions. After ethanol precipitation, total RNAs were harvested by centrifugation at 10,000 × g for 15 min, after which the concentration was determined by the Nano-300 Micro-Spectrophotometer (Allsheng Instruments Co. Ltd., Hangzhou, China). Total complementary DNA (cDNA) against mRNA was synthesized using 200 unit of Superscript II reverse transcriptase (Thermo Fisher Scientific Inc.). RT-qPCR was conducted using the cDNA template obtained (1 μL), along with 2 × Power SYBR Green (6 μL; Toyobo Life Science, Osaka, Japan) and specific primers (Additional file 1: Table S5). The cycle quantification value (Cq) was defined as described in the Livak and Schmittgen’s method [23].

### Statistical analysis

Statistical significance between the groups was analyzed by applying the One-way Analysis of Variance (ANOVA) (SPSS for Windows, Release 10.10, Standard Version, Chicago, IL, USA) followed by Tukey post hoc t-test for multiple comparisons. All values are presented as the means ± SD, and a *p* value (*p* < 0.05) is determined as statistically significant.

## Supplementary Information

**Additional file 1. Supplement table S1**. Alternation on the body and organ weight in BALB/cKorl mice with CT26-bearing tumor after treatment of cisplatin. The data are reported as the means ± SD. *, p < 0.05 relative to the No treated group. #, p < 0.05 compared to the Vehicle treated group.** Supplement table S2**. Alteration on the serum parameters in BALB/cKorl mice after cisplatin treatment. The data are reported as the means ± SD. *, p < 0.05 relative to the No treated group. #, p < 0.05 compared to the Vehicle treated group.** Supplement table S3**. Alteration on the blood parameters in BALB/cKorl mice after cisplatin treatment. The data are reported as the means ± SD. *, p < 0.05 relative to the No treated group. #, p < 0.05 compared to the Vehicle treated group.** Supplement table S4**. List of antibodies for Western blot analyses.** Supplement table S5**. Primer sequences for RT-qPCR.

## Data Availability

The datasets used and/or analyzed during the current study are available from the corresponding author on reasonable request.

## Abbreviations

AAALAC: Association for assessment and accreditation of laboratory animal care
ANOVA: Analysis of variance
BALB/cfCd: Claude BALB/c
Bax: Bcl-2-associated X protein
Bcl-2: B-cell lymphoma 2
Cas-3: Caspase-3
cDNA: Complementary DNA
CDX: Cell line-derived syngeneic tumor models
Cq: Cycle quantification value
FBS: Fetal bovine serum
H&E: Hematoxylin and eosin
HRP: Horseradish peroxidase
IF: Immunofluorescence
MDM2: Mouse double minute 2
MFDS: Ministry of food and drug safety
MMP2: Matrix metallopeptidase 2
NIFDS: National institute of food and drug safety evaluation
PKC: Protein kinase c
PNU-IACUC: Pusan national university-institutional animal care and use committee
RPMI 1640 Medium: Roswell park memorial institute 1640 Medium
SPF: Specific pathogen-free
TMEV: Theiler’s murine encephalomyelitis virus
VEGF: Vascular endothelial growth factor
