# Supplementary material for: Comparison of cisplatin-induced anti-tumor response in CT26 syngeneic tumors of three BALB/c substrains
Source: Lab Anim Res. 2021 Dec 8;37:33. doi: 10.1186/s42826-021-00110-3 (PMC8653566; doi:10.1186/s42826-021-00110-3)
Supplement: Supplementary file 1 — Additional file 1. Supplement table S1. Alternation on the body and organ weight in BALB/cKorl mice with CT26-bearing tumor after treatment of cisplatin. The data are reported as the means ± SD. *, p < 0.05 relative to the No treated group. #, p < 0.05 compared to the Vehicle treated group. Supplement table S2. Alteration on the serum parameters in BALB/cKorl mice after cisplatin treatment. The data are reported as the means ± SD. *, p < 0.05 relative to the No treated group. #, p < 0.05 compared to the Vehicle treated group. Supplement table S3. Alteration on the blood parameters in BALB/cKorl mice after cisplatin treatment. The data are reported as the means ± SD. *, p < 0.05 relative to the No treated group. #, p < 0.05 compared to the Vehicle treated group. Supplement table S4. List of antibodies for Western blot analyses. Supplement table S5. Primer sequences for RT-qPCR. [file 42826_2021_110_MOESM1_ESM.docx]

Additional file 1 : Supplement table S1. Alternation on the body and organ weight in BALB/cKorl mice with CT26-bearing tumor after treatment of cisplatin. The data are reported as the means ± SD. *, p < 0.05 relative to the No treated group. #, p < 0.05 compared to the Vehicle treated group.

| **Mice** | **BALB/cKorl** | | | | | **BALB/cA** | | | | **BALB/cB** | | | | | |
| --- | --- | --- | --- | --- | --- | --- | --- | --- | --- | --- | --- | --- | --- | --- | --- |
| Treatment  Group | No | Vehicle | LoC | MiC | HiC | No | Vehicle | LoC | MiC | HiC | No | Vehicle | LoC | MiC | HiC |
| Body weight  (mg) | 28.82  ±1.6 | 29.85  ±1.5 | 29.32  ±2.3 | 26.86  ±2.1 | 20.27  ±1.1 | 24.82  ±1.5 | 24.85  ±0.9 | 25.81  ±0.8 | 24.14  ±1.1 | 18.6  ±1.2 | 23.1  ±1.9 | 23.87  ±1.0 | 24.63  ±1.2 | 23.74  ±1.2 | 19.52  ±1.0 |
| Liver/Body  (%) | 5.87  ±0.3 | 5.59  ±0.5 | 5.64  ±0.2 | 5.20  ±0.2 | 4.37  ±0.3 | 5.33  ±0.3 | 5.04  ±0.5 | 4.99  ±0.3 | 5.11  ±0.3 | 3.94  ±0.3 | 4.58  ±0.3 | 4.51  ±0.3 | 4.66  ±0.4 | 4.51  ±0.4 | 3.75  ±0.3 |
| Kidney/Body  (%) | 1.65  ±0.0 | 1.7  ±0.1 | 1.78  ±0.1 | 1.69  ±0.1 | 1.76  ±0.1 | 1.67  ±0.1 | 1.68  ±0.2 | 1.69  ±0.1 | 1.67  ±0.1 | 1.68  ±0.1 | 1.54  ±0.1 | 1.52  ±.1 | 1.67  ±0.2 | 1.53  ±0.1 | 1.45  ±0.1 |
| Spleen/Body  (%) | 1.79  ±0.6 | 2.04  ±0.5 | 1.51  ±0.2 | 1.26  ±0.2 | 0.31  ±0.1 | 1.16  ±0.4 | 1.16  ±0.2 | 1.21  ±0.4 | 1.03  ±0.4 | 0.33  ±0.1 | 1.04  ±0.2 | 0.92  ±0.1 | 1.12  ±0.2 | 0.83  ±0.1 | 0.23  ±0.0 |
| Thymus/Body  (%) | 0.12  ±0.0 | 0.14  ±0.0 | 0.19  ±0.0 | 0.2  ±0.1 | 0.12  ±0.0 | 0.16  ±0.1 | 0.16  ±0.0 | 0.18  ±0.1 | 0.13  ±0.0 | 0.07  ±0.0 | 0.12  ±0.0 | 0.11  ±0.1 | 0.14  ±0.1 | 0.11  ±0.0 | 0.09  ±0.0 |

Additional file 2 : Supplement table S2. Alteration on the serum parameters in BALB/cKorl mice after cisplatin treatment. The data are reported as the means ± SD. *, p < 0.05 relative to the No treated group. #, p < 0.05 compared to the Vehicle treated group.

| **Mice** | **BALB/cKorl** | | | | | **BALB/cA** | | | | | **BALB/cB** | | | | |
| --- | --- | --- | --- | --- | --- | --- | --- | --- | --- | --- | --- | --- | --- | --- | --- |
| Treatment  Group | No | Vehicle | LoC | MiC | HiC | No | Vehicle | LoC | MiC | HiC | No | Vehicle | LoC | MiC | HiC |
| AST  (U/L) | 101.3  ±15 | 102.7  ±13 | 112.5  ±7 | 110.7  ±9 | 136.8  ±13 | 86.4  ±14 | 89.8  ±8 | 94.5  ±9 | 109.1  ±25 | 165.5  ±28 | 93.6  ±20 | 84.2  ±21 | 75.9  ±26 | 77.3  ±24 | 183.7±53 |
| ALT  (U/L) | 32.2  ±1.6 | 33.5  ±1.5 | 31.5  ±1.8 | 27.8  ±4.0 | 36.0  ±2.5 | 18.8  ±2.8 | 18.9  ±3.1 | 21.0  ±2.2 | 25.7  ±3.9 | 30.8  ±3.3 | 42.0  ±15 | 34.1  ±12 | 33.5  ±12 | 32.6  ±17 | 71.8  ±20 |
| GGT  (U/L) | 0.00  ±0.0 | 0.00  ±0.0 | 0.00  ±0.0 | 0.00  ±0.0 | 0.00  ±0.0 | 0.00  ±0.0 | 0.00  ±0.0 | 0.00  ±0.0 | 0.11  ±0.3 | 0.33  ±0.5 | 0.00  ±0.0 | 0.00  ±0.0 | 0.00  ±0.0 | 0.00  ±0.0 | 0.40  ±0.5 |
| ALP  (g/mL) | 40.7  ±5.8 | 40.0  ±2.8 | 43.3  ±2.5 | 41.0  ±3.3 | 62.3  ±3.3 | 43.8  ±8.8 | 42.0  ±5.5 | 54.3  ±4.5 | 46.8  ±8.5 | 93.0  ±17 | 51.7  ±6.7 | 51.4  ±10 | 50.8  ±14 | 46.1  ±12 | 120.3  ±11 |
| LDH  (U/L) | 1085  ±54 | 954  ±139 | 1018  ±166 | 987  ±147 | 868  ±83 | 354  ±53 | 591  ±59 | 745  ±67 | 766  ±68 | 572  ±54 | 420  ±79 | 306  ±84 | 311  ±96 | 394  ±113 | 395  ±136 |
| Ca  (mg/dL) | 8.6  ±0.7 | 8.4  ±0.8 | 9.3  ±0.7 | 8.9  ±0.5 | 8.5  ±0.6 | 9.3  ±0.9 | 9.3  ±0.9 | 8.9  ±0.6 | 9.1  ±0.7 | 8.8  ±0.2 | 7.5  ±0.8 | 7.6  ±0.8 | 7.3  ±0.9 | 7.9  ±0.6 | 7.5  ±0.9 |

Additional file 3 : Supplement table S3. Alteration on the blood parameters in BALB/cKorl mice after cisplatin treatment. The data are reported as the means ± SD. *, p < 0.05 relative to the No treated group. #, p < 0.05 compared to the Vehicle treated group.

| **Mice** | **BALB/cKorl** | | | | | **BALB/cA** | | | | | **BALB/cB** | | | | |
| --- | --- | --- | --- | --- | --- | --- | --- | --- | --- | --- | --- | --- | --- | --- | --- |
| Treatment  Group | No | Vehicle | LoC | MiC | HiC | No | Vehicle | LoC | MiC | HiC | No | Vehicle | LoC | MiC | HiC |
| WBC  (x10^3^ cells/μl) | 4.56  ±1.4 | 4.71  ±0.3 | 5.92  ±1.6 | 4.13  ±0.9 | 5.10  ±1.1 | 6.55  ±1.5 | 6.52  ±1.5 | 7.47  ±0.8 | 5.16  ±1.5 | 4.26  ±1.2 | 3.14  ±0.7 | 4.56  ±1.2 | 3.97±1.5 | 4.68  ±1.3 | 3.36  ±0.7 |
| LYM  (x10^3^ cells/μl) | 1.97  ±0.6 | 2.00  ±0.8 | 1.81  ±0.5 | 1.62  ±0.5 | 1.50  ±0.2 | 2.69  ±0.8 | 3.00  ±0.4 | 3.50  ±0.9 | 2.21  ±0.5 | 1.13  ±0.3 | 1.02  ±0.4 | 1.71  ±0.6 | 1.54  ±0.5 | 2.14  ±0.5 | 1.19  ±0.5 |
| NEU  (x10^3^ cells/μl) | 2.22±1.0 | 2.39  ±0.8 | 3.59  ±1.3 | 2.16  ±0.6 | 3.06  ±0.9 | 3.27  ±1.5 | 2.92  ±1.5 | 3.38  ±0.9 | 2.96  ±0.9 | 2.68  ±0.9 | 1.88  ±0.6 | 2.48  ±0.5 | 1.83  ±0.5 | 2.03  ±0.5 | 1.87  ±0.3 |
| RBC  (x10^6^ cells/μl) | 9.17±0.8 | 9.02  ±1.6 | 8.65  ±0.5 | 8.76  ±1.1 | 10.5  ±1.0 | 8.6  ±1.3 | 8.5  ±1.1 | 9.06  ±0.4 | 8.00  ±0.8 | 10.32  ±0.7 | 8.51  ±1.1 | 9.01  ±0.3 | 8.28  ±1.1 | 8.77  ±0.9 | 11.03  ±1.0 |
| HGB  (g/dL) | 12.77±1.3 | 12.44  ±2.3 | 12.20  ±0.5 | 12.44  ±1.5 | 14.48  ±1.5 | 11.29  ±1.3 | 11.70  ±1.3 | 12.27  ±0.7 | 11.42  ±0.9 | 14.56  ±1.5 | 12.48  ±0.3 | 12.20  ±0.4 | 11.90  ±0.9 | 12.16  ±1.1 | 15.10  ±2.0 |
| HCT  (%) | 41.29  ±2.1 | 39.98  ±5.0 | 39.77  ±2.5 | 41.81  ±3.4 | 43.99  ±4.0 | 40.28  ±4.3 | 37.89  ±3.7 | 42.02  ±1.8 | 37.59  ±1.9 | 45.83  ±3.1 | 35.22  ±1.1 | 35.50  ±1.3 | 34.97  ±1.7 | 35.38  ±2.0 | 36.12  ±2.5 |
| MCV  (fL) | 45.17  ±2.4 | 44.63  ±2.9 | 46.00  ±2.0 | 48.00  ±3.2 | 42.00  ±1.5 | 47.00  ±3.2 | 45.00  ±2.7 | 46.44  ±1.1 | 47.33  ±3.5 | 44.43  ±0.8 | 39.20  ±0.8 | 39.43  ±0.8 | 40.67  ±1.2 | 40.50  ±1.4 | 37.67  ±1.5 |
| MCH  (pg) | 13.92  ±0.4 | 13.79  ±0.6 | 15.13  ±0.5 | 14.19  ±0.6 | 13.77  ±0.4 | 14.36  ±1.0 | 13.88  ±0.5 | 13.51  ±0.6 | 14.34  ±0.6 | 14.01  ±0.6 | 13.63  ±0.4 | 13.56  ±0.2 | 13.73  ±0.4 | 13.80  ±0.3 | 13.67  ±0.7 |
| MCHC  (g/dL) | 30.90  ±2.2 | 30.94  ±1.9 | 30.71  ±1.1 | 29.66  ±2.0 | 32.95  ±0.9 | 30.64  ±2.5 | 30.85  ±1.5 | 29.16  ±1.3 | 30.38  ±1.4 | 31.70  ±1.9 | 35.24  ±0.9 | 34.39  ±0.4 | 33.79  ±1.3 | 34.33  ±0.9 | 36.17  ±0.1 |
| RDW  (%) | 19.97  ±1.0 | 20.91  ±1.2 | 20.40  ±0.7 | 20.70  ±0.5 | 21.17  ±3.7 | 21.17  ±1.9 | 20.29  ±1.8 | 21.19  ±2.3 | 20.52  ±1.2 | 20.36  ±0.9 | 19.26  ±0.5 | 19.53  ±0.4 | 19.83  ±0.6 | 20.22  ±0.5 | 19.43  ±0.3 |
| PLT  (x10^3^ cells/μl) | 465.6  ±120 | 405.6  ±133 | 329.1  ±44 | 395  ±63 | 364  ±94 | 330  ±65 | 390  ±53 | 452  ±78 | 396  ±88 | 235  ±55 | 391  ±57 | 400  ±58 | 403  ±60 | 406  ±59 | 205±53 |
| MPV  (fL) | 6.22  ±0.2 | 6.13  ±0.1 | 6.23  ±0.5 | 6.11  ±0.2 | 6.03±0.4 | 6.11  ±0.3 | 5.98  ±0.2 | 5.98  ±0.2 | 5.98  ±0.2 | 6.07  ±0.4 | 6.04  ±0.2 | 5.74  ±0.3 | 5.68  ±0.2 | 5.79  ±0.3 | 5.50  ±0.2 |

Additional file 4 : Supplement table S4. List of antibodies for Western blot analyses

| Name | Cat. No. | Company, City, State, Country |
| --- | --- | --- |
| Anti-Bax [E63] | ab32503 | Abcam, Cambridge, UK |
| Anti-Bcl-2 | PA5-20069 | Thermo Fisher Scientific, Bothell Washington, USA |
| Anti-Cas-3 | 9662S | Cell Signaling, Danvers, MA, USA |
| Anti-actin | 4967S | Cell Signaling, Danvers, MA, USA |
| Anti-VEGF | 500-P131 | PeproTech Ltd., London, UK |
| Anti-MMP-2 (H-76) | SC-10736 | Santa Cruz Biotechnology, Inc. Santa Cruz, CA, USA |
| Anti-p53 | 10442-1-AP | Proteintech, Rosemont, IL 60018, USA |
| Anti-p27 | 2552S | Cell signaling, Danvers, MA, USA |
| Anti-Ki67 | NB500-170 | Novusbio, Centennial, Colorado, USA |

Additional file 5 : Supplement table S5. Primer sequences for RT-qPCR

| Primer name | Sequence (from 5’ to 3’) | Product size (bp) |
| --- | --- | --- |
| IL-1β  Forward  Reverse | CTG TCC TGA TGA GAG CAT CCA GCT TC  GTT GCT TGG TTC TCC TTG TAC AAA GCT C | 388 |
| IL-6  Forward  Reverse | CTC TCT GCA AGA GAC TTC CAT CCA G  GCT ATG GTA CTC CAG AAG ACC AGA GG | 357 |
| IL-10  Forward  Reverse | CTC TTA CTG ACT GGC ATG AGG ATC AG  CTA TGC AGT TGA TGA AGA TGT CAA ATT C | 475 |
| β-actin  Forward  Reverse | TGG AAT CCT GTG GCA TCC ATG AAA C  TAA AAC GCA GCT CAG TAA CAG TCC G | 349 |
